# Zika virus displacement by a chikungunya outbreak in Recife, Brazil

**DOI:** 10.1371/journal.pntd.0006055

**Published:** 2017-11-06

**Authors:** Tereza Magalhaes, Cynthia Braga, Marli T. Cordeiro, Andre L. S. Oliveira, Priscila M. S. Castanha, Ana Paula R. Maciel, Nathalia M. L. Amancio, Pollyanne N. Gouveia, Valter J. Peixoto-da-Silva, Thaciana F. L. Peixoto, Helena Britto, Priscilla V. Lima, Andreza R. S. Lima, Kerstin D. Rosenberger, Thomas Jaenisch, Ernesto T. A. Marques

**Affiliations:** 1 Laboratory of Virology and Experimental Therapeutics, Aggeu Magalhaes Institute (Instituto Aggeu Magalhães-IAM), Oswaldo Cruz Foundation (Fundação Oswaldo Cruz-FIOCRUZ), Recife, Brazil; 2 Arthropod-borne and infectious Diseases Laboratory (AIDL), Department of Microbiology, Immunology and Pathology, Colorado State University (CSU), Fort Collins, United States of America; 3 Department of Parasitology, IAM, FIOCRUZ, Recife, Brazil; 4 Institute of Integral Medicine Professor Fernando Figueira (Instituto de Medicina Integral Professor Fernando Figueira-IMIP), Recife, Brazil; 5 Statistics and Geoprocessing Laboratory, IAM, FIOCRUZ, Recife, Brazil; 6 Faculty of Medical Science, University of Pernambuco (Universidade de Pernambuco-UPE), Recife, Brazil; 7 Urgent Health Care Unit (Unidade de Pronto Atendimento-UPA) of Paulista, IMIP, Paulista, Brazil; 8 Section Clinical Tropical Medicine, Department of Infectious Diseases, Heidelberg University Hospital, Heidelberg, Germany; 9 German Centre for Infection Research (DZIF), partner site Heidelberg, Heidelberg, Germany; 10 Center for Vaccine Research, University of Pittsburgh, Pittsburgh, United States of America; Center for Disease Control and Prevention, UNITED STATES

## Abstract

**Background:**

Several arboviruses, including dengue virus (DENV), Zika virus (ZIKV) and chikungunya virus (CHIKV), transmitted by *Aedes* mosquitoes, circulate in northeast Brazil. Diseases caused by these viruses are of great public health relevance, however, their epidemiological features in areas where the three viruses co-circulate are scarce. Here, we present analyses of molecular and serological diagnostics in a prospective study of acute febrile patients recruited from May 2015 to May 2016 in Recife, Brazil.

**Methods:**

Two hundred sixty-three acute febrile patients with symptoms suggestive of an arboviral disease who attended an urgent heath care clinic in the Recife Metropolitan Region in northeast Brazil were enrolled. Acute and convalescent blood samples were collected and tested using molecular and serological assays for infection with DENV, ZIKV and CHIKV.

**Results:**

Quantitative real-time reverse-transcriptase polymerase chain reactions (qRTPCR) performed on acute phase sera detected no patients positive for DENV, but 26 (9.9%) positive for ZIKV and 132 (50.2%) positive for CHIKV. There were a few suspected and only one confirmed dengue case. Specific serological assays for ZIKV and CHIKV confirmed the qRTPCR data. Analyses of DENV IgM and IgG ELISAs in the context of qRTPCR results suggested high levels of cross reactive antibodies in ZIKV-positive samples. Results from neutralization assays highly corroborated those from qRTPCR and ZIKV ELISA, indicating very few positive DENV cases. ZIKV infections were temporally clustered in the first months of the study and started to decrease concomitantly with an increase in CHIKV infections in August 2015. The proportion of CHIKV infections increased significantly in September 2015 and remained high until the end of the study period, with an average of 84.7% of recruited patients being diagnosed from August 2015 to May 2016. ZIKV infections exhibited a female bias and the cases were spread over the study site, while CHIKV cases had a male bias and were spatially clustered in each month.

**Conclusions:**

In 2015–2016 in the Recife Metropolitan Region, we detected the tail end of a Zika epidemic, which was displaced by a chikungunya epidemic. Few dengue cases were identified despite a high number of official dengue notifications in the area during this period. We show here important epidemiological features of these cases.

## Author summary

Infection with Zika (ZIKV) and chikungunya (CHIKV) viruses can lead to severe symptoms or outcomes. These viruses are transmitted by the same mosquito vectors that transmit dengue virus (DENV) and the symptoms of all three diseases are similar, thus, it may be difficult for health professionals to diagnose them properly. This study was part of larger study aimed at improving the diagnosis of these diseases. We present data on a patient cohort from Recife, Brazil in which the majority of patients were infected with ZIKV or CHIKV. We show that when Zika cases started to decrease, chikungunya cases appeared and increased rapidly, remaining high until the end of the study. We also highlight the high levels of cross-reactivity on dengue serological assays with ZIKV-positive samples, emphasizing the importance of choosing appropriate tests in areas with simultaneous circulation of arboviruses. Other important epidemiological data are shown and discussed, such as the differential proportion of women and men infected with ZIKV or CHIKV, and the identification of strong hotspots of chikungunya cases. These data may help on the development of control and prevention interventions against arboviral diseases.

## Introduction

The State of Pernambuco in northeast Brazil has been endemic for dengue virus (DENV) since at least 1995, with all four serotypes (DENV1-DENV4) circulating in the region [1–3]. In 2015, Zika (ZIKV) and chikungunya (CHIKV) viruses were also detected in Pernambuco [3–5]. The co-circulation of these viruses within a geographic region poses a great challenge to health authorities as many signs and symptoms are shared among symptomatic patients, varying only in severity level and time of onset [5], which makes differential clinical diagnosis difficult when sensitive and specific diagnostic tests are not available.

Reported cases of Zika were sporadic until 2007, when an outbreak occurred on Yap Island in the Pacific Ocean [6] and was followed by outbreaks in 2013–2014 in French Polynesia [7, 8]. Then, in April of 2015, ZIKV was detected in northeast Brazil and quickly spread to the rest of the country and to the Americas [9, 10]. In October of 2015, a milestone in Zika epidemiology occurred when the first association of microcephaly in babies born of mothers infected with ZIKV during pregnancy was suggested by the Microcephaly Epidemic Research Group (MERG) [11, 12]. The estimated number of ZIKV cases in Brazil in 2015 ranges from 497,593 to 1,482,701, of which an estimated 34,579 to 81,303 cases were from Pernambuco [13]. However, epidemiological data strengthening these estimates are needed.

CHIKV similarly has disseminated globally after it caused large epidemics in the coastal region of Kenya, then spread further to several islands in the Indian Ocean and eventually to other regions in Asia, Africa and parts of Europe [14, 15]. After spreading in the Pacific region in 2013, CHIKV was detected in the Caribbean [15]. In Brazil, the first outbreak of CHIKV infections occurred in Bahia State in 2014 [16, 17]. In 2016, there were 271,284 of notified chikungunya cases throughout the country, a number significantly higher compared to 2015 [18]. Disease caused by CHIKV often causes severe, debilitating short- and long-term arthralgia, among other symptoms [19]. Still, very few reports on CHIKV epidemiological features in Brazil are available.

The present study was part of the IDAMS (International Research Consortium on Dengue Risk Assessment, Management and Surveillance) observational prospective multicenter study [20, 21]. The aim of the IDAMS study was to gather extensive clinical and laboratory data of patients presenting with acute undifferentiated febrile illness to evaluate prognostic indicators of a severe course of disease and to differentiate dengue from other febrile illness based on clinical and readily available laboratory markers in the early phase of the illness.

Not surprisingly, ZIKV- and CHIKV-infected patients fitting the IDAMS study’s inclusion criteria of undifferentiated febrile illness within the first 72 hours of sign/symptom onset were enrolled in the Recife cohort. Detailed clinical and epidemiological data on ZIKV and CHIKV infections are critical to guide control and prevention interventions. Here, we present the epidemiological features of these cases. The clinical features of the diseases caused by the three arboviruses (ZIKV, CHIKV and DENV) currently circulating in Latin America will be presented elsewhere.

## Methods

### Study design, setting and participants

The local study protocol followed the multicenter study protocol as reported by Jaenisch et al. [21]. The primary aim of IDAMS was to identify, in a prospective cohort, simple laboratory or clinical markers that should be able to: 1) help distinguish dengue from other arboviral diseases and 2) help quickly identify patients in the early stages of disease who may progress to severe dengue.

Considering these aims, the protocol was focused on symptomatic patients at the very early clinical phase of the disease. The inclusion criteria were: age ≥ 5 years; fever or history of fever for ≤ 72 h; clinical symptoms consistent with possible dengue, i.e., suspicion of dengue and/or undifferentiated fever in a patient from a dengue endemic area; considered by the treating physician to be suitable for outpatient care at the time of study enrolment, i.e., no signs of severe disease; and written informed consent. The exclusion criteria were: localizing features suggesting an alternative diagnosis, e.g., pneumonia, otitis, etc., and judgement by the physician that the patient was unlikely to attend daily follow up, e.g., due to travelling distance from the clinic.

The number of study visits required for each patient was dependent on the presence of fever. Visits had to occur daily while fever persisted, and for two days (48 h) after fever ceased (defervescence period), in addition to a convalescent visit 10–30 days after fever onset. Thus, a minimum of three acute illness visits and one convalescent visit per patient was required for study completion.

In all visits (initial and follow-ups), venous blood was collected and clinical evaluation was performed. The clinical research form was a 19-page document that included questions on demographics, medical history, vital signs and symptoms, clinical evaluation, data on hospitalization (if applicable), and a summary form. Clinical data will not be presented here as they will be part of the overall study results involving all sites.

The local study population were people presenting with illness at a rapid-access health care unit (Unidade de Pronto Atendimento-UPA) in the city of Paulista in the Recife Metropolitan Region (RMR), State of Pernambuco, Brazil, between May 2015 and May 2016. The UPAs are health care facilities assisting the surrounding communities to minimize the burden of high patient numbers in hospitals. The unit in Paulista (UPA-Paulista) offers a medical clinic as well as pediatric and dentistry specialties and services including X-ray, electrocardiography, laboratory tests and observation units. It operates 24 hours a day, seven days a week, and approximately 350 patients are seen per day.

### Biobank

Blood samples were collected to perform biochemical (albumin, creatinine, creatinine kinase, alanine aminotransferase-ALT and aspartate aminotransferase-AST) and hematological (whole blood count) tests as well as specific molecular and serological tests for ZIKV, CHIKV and DENV. Hematological and biochemical exams were performed by a local clinical diagnostic laboratory (Cerpe Diagnósticos, DASA) that collected patient samples daily at the UPA. The results of the blood tests will not be presented here. Samples used for lab experiments were registered, processed (centrifuged at 1500xg for 5 min), aliquoted, stored at -80°C, and mapped.

### Molecular assays

Quantitative real-time PCR (qRTPCR) was performed for the detection of DENV, ZIKV or CHIKV RNA separately in the first acute sample of patients (n = 263). For this, RNA was extracted from plasma using QIAamp Viral RNA Mini Kit (Qiagen, Valencia, U.S.) according to the manufacturer’s instructions, and a small aliquot was used for qRTPCR with primers specific for each of the viruses (Table 1). Reactions were prepared using the GoTaq Probe 1-Step RT-qPCR System (Promega, Madison, U.S.) and were run in an Applied Biosystems 7500 Real-Time PCR System. Protocols were slightly modified from previously reported assays [22–25]. Positive controls were viruses extracted from cell culture, and the negative control was water.

**Table 1:** Quantitative real-time PCR (qRTPCR) primers and probes for Zika virus (ZIKV), dengue virus (DENV) and chikungunya virus (CHIKV).

Primer	Sequence (5’-3’)	References
ZIKV Fwd	CCGCTGCCCAACACAAG	
ZIKV Rev	CCACTAACGTTCTTTTGCAGACAT	[22]
ZIKV Probe	**VIC**-AGCCTACCTTGACAAGCAGTCAGACACTCAA-**BHQ1**	
DENV-G Fwd	AAGGACTAGAGGTTAGAGGAGACCC	
DENV-G Rev	CGTTCTGTGCCTGGAATGATG	[24, 25]
DENV-G Probe	**FAM**-AACAGCATATTGACGCTGGGAGAGACCAGA-**BHQ1**	
CHIKV Fwd	TCACTCCCTGTTGGACTTGATAGA	
CHIKV Rev	TTGACGAACAGAGTTAGGAACATACC	[23]
CHIKV Probe	**FAM**-AGGTACGCGCTTCAAGTTCGGCG-**BHQ1**	

### Serological assays

#### Enzyme-linked immunosorbent assay (ELISA)

For dengue serology, Panbio Dengue IgM or IgG Capture ELISAs (Alere, Waltham, U.S.) were used to detect DENV-specific IgM and IgG antibodies, respectively, in patient sera following the manufacturer’s instructions. Both acute (n = 263) and convalescent (n = 191) samples were assayed. In addition, to check the status of previous exposure to DENV, acute samples that tested negative in the DENV IgG capture ELISA were assayed by the Panbio Dengue IgG Indirect ELISA (Alere).

Convalescent samples were assayed for ZIKV-specific IgM antibody detection using the MAC-ELISA protocol from the U.S. Centers for Diseases Control and Prevention (CDC) [23, 26], which uses ZIKV and DENV antigens in parallel.

CHIKV-specific IgM was assayed in the convalescent samples using an anti-chikungunya virus ELISA IgM kit (Euroimmun, Lubeck, Germany), following the manufacturer’s protocol.

Lastly, DENV non-structural protein 1 (NS1) was measured in the first acute samples of all patients using the Platelia Dengue NS1 Ag kit (Bio-Rad, Marnes-la-Coquette, France), according to the manufacturer’s instructions.

#### Plaque Reduction and Neutralization Test (PRNT)

DENV1-4 and ZIKV-specific neutralizing antibodies were assessed by PRNTs, following a modified protocol described in detail elsewhere [27]. PRNTs were performed with Vero cells using virus strains isolated in the study setting: DENV-1 (BR-PE/97-42735); DENV-2 (BR-PE/95-3808); DENV-3 (BR-PE/02-95016); DENV-4 (BR-PE/12-008) and ZIKV (BR-PE243/2015). The cut-off for PRNT positivity was defined based on a 50% reduction in plaque counts (PRNT_50_). Samples were considered positive when neutralizing antibody levels were ≥1:100 for ZIKV and ≥1:20 against at least one DENV serotype. ZIKV and DENV serotype-specific antibody titers were estimated using a four-parameter non-linear regression. Acute and convalescent samples were assayed in parallel and a convalescent/acute titer ratio >4 was indicative of an acute infection.

PRNTs were performed with: 1) sera from all patients with a confirmed or suspected ZIKV infection that had a convalescent sample; 2) sera from seventeen patients (randomly selected among those that had a convalescent sample) with a confirmed CHIKV infection; and 3) sera from twenty-nine out of seventy-seven patients that were not considered as having an infection with ZIKV or CHIKV.

### Diagnosis

Patients were considered positive for any of the viruses (DENV, ZIKV or CHIKV) when qRTPCR with the respective specific primers was positive. Samples with equivocal qRTPCR results (when only one of the duplicates had a positive cycle threshold value) were considered negative.

Patients were considered to have a recent probable infection (suspected case) with ZIKV or CHIKV if only the ZIKV- or CHIKV-specific IgM assay was positive, respectively, but not the qRTPCR. Suspected Zika cases were further assayed through PRNT and were discarded if the PRNT results did not meet those indicative of an acute infection with ZIKV.

For DENV, besides qRTPCR results, patients were also considered positive if the acute sample was positive for DENV NS1. DENV diagnosis based on the interpretation of DENV-specific antibodies using the Panbio capture tests was not performed due to the high cross-reactivity observed among patients infected with ZIKV. Patients were considered as having a suspected DENV infection if the PRNT results were indicative of such. A previous exposure to DENV was indicated if the acute sample tested positive for anti-DENV IgG using the Panbio capture or indirect ELISAs.

### Spatiotemporal distribution and density-based cluster analysis of ZIKV and CHIKV cases

For spatiotemporal analysis, the address reported in a patient’s file was transformed into spatial data and a geographic database was then generated using QGIS version 2.11. The SIRGAS 2000 geodetic system (available at http://www.ibge.gov.br/english/geociencias/geodesia/pmrg/faq.shtm#1) was used to generate the maps. Spatially distributed ZIKV and CHIKV cases were superimposed into a satellite image of the study area, taken with Landsat-8 satellite and made publicly available at the Brazilian National Institute for Space Research webpage. Finally, Kernel density-based analysis was performed with ZIKV and CHIKV cases. These maps were constructed for the purposes of this manuscript only and have not been previously published.

### Statistical tests

Unpaired *T*-test was used to compare the differences in qRTPCR Ct values between ZIKV- and CHIKV-infected patients. Paired *T*-test was performed to compare the fold difference in Panbio units between acute and convalescent samples in the DENV Capture ELISAs (IgM and IgG). This comparison was performed with samples from patients that were diagnosed with ZIVK or CHIKV infections. GraphPad Prism 7 was used for these tests.

To compare the proportions of women and men among ZIKV- and CHIKV-infected groups, exact 95% confidence intervals for proportions were calculated in Stata/IC 13.1 (College Station, Texas: StataCorp LP). Tests on the equality of proportions were performed using Stata’s “prtest” command.

### Ethics statement

The study protocol (which included the IDAMS study protocol and adapted components of the local study) was reviewed and approved by the local ethics committee on human research at the Instituto Aggeu Magalhães-Fundação Oswaldo Cruz (IAM-FIOCRUZ) (approval ID 28309414.9.0000.5190), the local ethics committee on human research at the Instituto de Medicina Integral Professor Fernando Figueira (IMIP) (approval ID 28309414.9.3001.5201) and the national ethics committee on human research of Brazil (approval ID 15580013.5.1001.5534). All adult subjects provided informed consent, and a parent or guardian of any child participant provided informed consent on their behalf. The informed consents were written, and all patient data analyzed was anonymized.

## Results

### Patient recruitment

**Fig 1:**
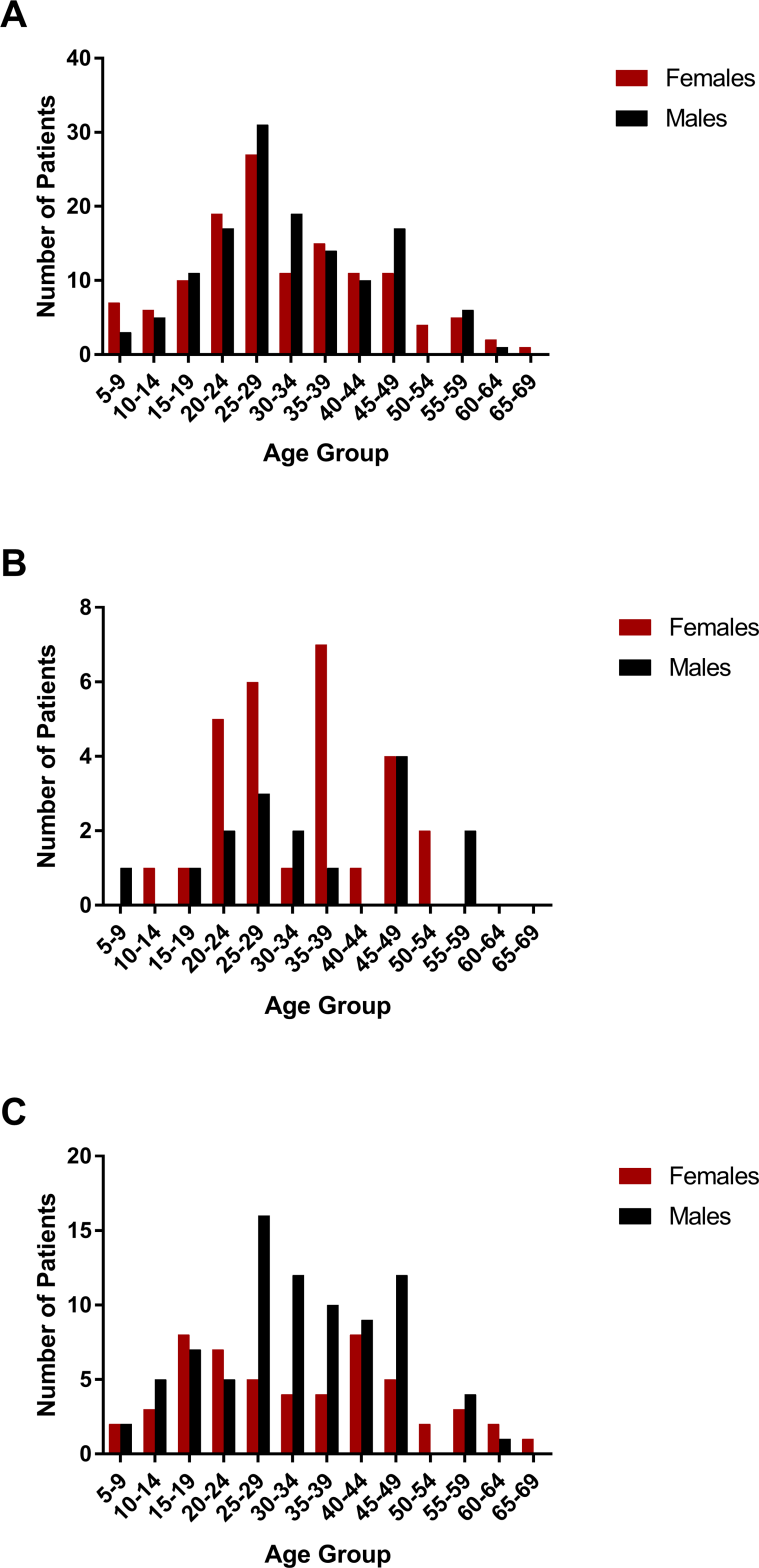
Females and males in different age groups. (A) Number of females and males in different age groups among all recruited patients. (B) Number of females and males in different age groups among patients infected with Zika virus (ZIKV). (C) Number of females and males in different age groups among patients infected with chikungunya virus (CHIKV).

From May 2015 to May 2016, 263 patients who fulfilled the study protocol inclusion criteria were enrolled in the study at UPA-Paulista. There was no significant difference in the percentages of females (129; 49.0%) and males (134; 51.0%) enrolled. The age of participants ranged from 6–67 years (median, 29; interquartile range, 23–40). Fig 1 depicts the gender distribution among the age groups of enrolled patients.

A total of 938 medical visits were documented, with an average of 3.6 visits per patient, which typically included the enrolment exam and two or three follow-up visits. The monthly pattern of patient recruitment varied over the study period and tended to decline towards the end (Table 2). The number of patients who completed the study protocol was 178 (67.7%), while 85 (32.3%) patients did not complete the study (loss due to failure to follow-up). There was no significant difference between men and women in regards to study completion (48.9% men versus 51.1% women). The number of patients who had a convalescent sample collected was 191. The mean time for the convalescent visit was 15 days after recruitment (interquartile range, 11–26). The number of hospitalizations among enrolled patients was 3.

**Table 2:** Patients with suspicion of an arboviral infection recruited at an urgent healthcare unit in the Recife Metropolitan Region, northeast Brazil, from May 2015 to May 2016.

Month/Year	Total number of patients recruited	Patients with complete follow-up (%)	Loss to follow-up (%)
May/2015	25	14 (56.0)	11 (44.0)
June/2015	44	31 (70.5)	13 (29.5)
July/2015	22	13 (59.1)	9 (40.9)
August/2015	20	13 (65.0)	7 (35.0)
September/2015	32	25 (78.1)	7 (21.9)
October/2015	15	14 (93.3)	1 (6.7)
November/2015	24	13 (54.2)	11 (45.8)
December/2015	12	7 (58.3)	5 (41.7)
January/2016	15	10 (66.7)	5 (33.3)
February/2016	18	16 (88.9)	2 (11.1)
March/2016	17	11 (64.7)	6 (35.3)
April/2016	12	8 (66.7)	4 (33.3)
May/2016	7	3 (42.9)	4 (57.1)
Total	263	178 (67.7)	85 (32.3)

**Fig 2:**
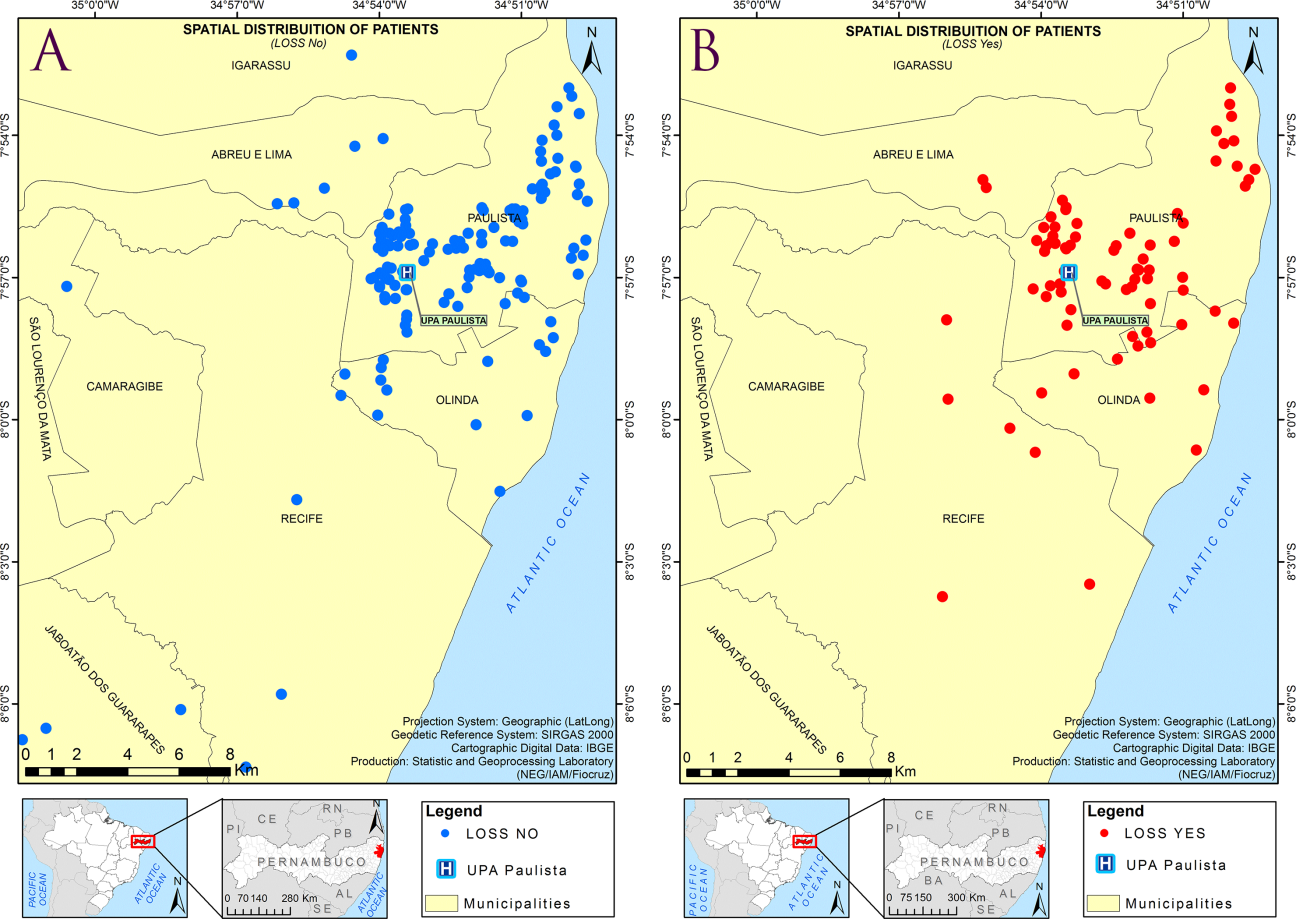
Spatial distribution of recruited patients in the study area (Recife Metropolitan Region), from May 2015 to May 2016. (A) Patients that completed the study. (B) Patients who were considered as loss. The geodetic reference system SIRGAS2000 (Geocentric Reference System for the Americas) was the coordinated system used to represent geometric or physical terrestrial characteristics (http://www.ibge.gov.br/english/geociencias/geodesia/pmrg/faq.shtm#1).

The majority of patients (81.4%) lived in the city of Paulista (where the health center is located), whereas 18.6% lived in the surrounding cities within the Recife Metropolitan Region. There was no difference in the spatial distribution of patients that completed the study and those that were considered as loss to follow-up (Fig 2).

Among all of the patients, 42 (15.9%) and 137 (52.1%) were considered to be infected (confirmed or suspected) with ZIKV and CHIKV, respectively.

### Zika infection

Out of the 263 acute samples tested, 26 (9.9%) were positive for ZIKV through qRTPCR; these patients were considered as having a confirmed infection with ZIKV. As for ZIKV serology, 36 out of 191 (18.8%) convalescent samples were positive in the IgM MAC-ELISA. Of these 36, 12 had the acute sample positive for ZIKV through qRTPCR. The remaining 24 were considered as having a suspected infection with ZIKV, and 22 were assayed for PRNT; if the PRNT result was not indicative of an acute infection with ZIKV, the case was discarded as suspected.

In the PRNTs, patients considered as confirmed for ZIKV infection based on qRTPCR had high titers of neutralizing antibodies against ZIKV confirmatory of an acute infection (PRNT_50_ titers in early convalescent sample ranged from 1,097 to 31,059) (Table 3).

**Table 3:** Molecular and serological results of patients with a confirmed Zika virus (ZIKV) infection.

Patient ID	Days post symptoms onset	Lab diagnostic assays													
		qRT-PCR			Serology						PRNT_50_				
					IgM-capture ELISA			IgM/IgG capture ELISA†		IgG-indirect ELISA§+					
		ZIKV	DENV	CHIKV	ZIKV IgM‡	DENV IgM‡	CHIKV IgM*	DENV IgM	DENV IgG	DENV IgG	DENV1	DENV2	DENV3	DENV4	ZIKV
62–0008	1	Pos	Neg	Neg	-	-	-	2.9	3.8	1.9	<20	<20	<20	20	<100
	15	-	-	-	5.2	1.5	0.2	25.3^POS^	64.6 ^POS^	-	<20	<20	806.7	896.7	6651
62–0016	3	Pos	Neg	Neg	-	-	-	10.2^EQU^	6.3	3.3	<20	<20	966.9	689.7	<100
	16	-	-	-	7.1	1.1	0.6	19.2 ^POS^	47.7 ^POS^	-	<20	<20	6300	11067	1087
62–0017	1	Pos	Neg	Neg	-	-	-	4.2	1.3	0.9	24.8	<20	<20	365.8	<100
	13	-	-	-	15.5	1.9	0.2	46.3 ^POS^	48.1 ^POS^	-	25.9	<20	946	1132	5698
62–0020	3	Pos	Neg	Neg	-	-		2.3	1.1	0.03	<20	<20	<20	<20	<100
	14	-	-	-	14.6	1.3	0.2	4.2	0.4	-	<20	<20	<20	<20	1227
62–0023	3	Pos	Neg	Neg	-	-	-	7.4	31.4 ^POS^	-	<20	<20	421.8	1674	<100
	13	-	-	-	8.0	1.5	0.2	24.9 ^POS^	64.8 ^POS^	-	66.9	44.9	1519	7580	31059
62–0032	2	Pos	Neg	Neg	-	-	-	2.6	11.2	3.3	<20	<20	124.9	91.7	<100
	15	-	-	-	14.4	1.6	0.3	16.7 ^POS^	65.3 ^POS^	-	<20	26.8	1451	1516	8974
62–0035	3	Pos	Neg	Neg	-	-		5.8	0.5	0.01	<20	<20	<20	<20	<100
	16	-	-	-	17.6	1.3	0.5	4.3	0.5	-	<20	<20	<20	<20	11569
62–0048	1	Pos	Neg	Neg	-	-	-	6.2	5.2	2.9	<20	<20	<20	<20	<100
	13	-	-	-	7.6	2.2	0.9	124.7 ^POS^	61.7 ^POS^	-	<20	<20	1710	812.6	5556
62–0050	2	Pos	Neg	Neg	-	-	-	8.0	7.0	3.3	78	53.9	106.8	48.5	171.2
	18	-	-	-	13.6	10.8	0.3	120.4 ^POS^	52.5 ^POS^	-	613.9	128.1	1395	965.3	5370
62–0056	5	Pos	Neg	Neg	-	-	-	11.1 ^POS^	2.3	3.0	<20	<20	<20	31.2	286.6
	24	-	-	-	14.3	0.9	0.1	10.1^EQU^	30.6 ^POS^	-	<20	<20	140.5	1321	4743
62–0060	2	Pos	Neg	Neg	-	-	-	4.5	1.7	1.4	<20	29.3	<20	<20	<100
	13	-	-	-	16.7	0.8	0.4	13.3 ^POS^	58.0 ^POS^	-	<20	54.8	215.2	653.1	4445
62–0083	0	Pos	Neg	Neg	-	-	-	2.7	0.2	0.02	<20	<20	<20	<20	<100
	29	-	-	-	5.2	0.9	0.4	2.7	0.2	-	<20	<20	<20	<20	1218
62–0095	1	Pos	Neg	Neg	-	-	-	3.8	9.3	3.2	<20	<20	58.8	47.6	<100
	11	-	-	-	10.7	1.2	0.2	11.1 ^POS^	49.8 ^POS^	-	<20	<20	1455	698.1	29566

Abbreviations: qRT-PCR, quantitative real time polymerase-chain reaction; ELISA, enzyme-linked immunosorbent assay; IgM, immunoglobulin M; IgG, immunoglobulin G; ZIKV, Zika virus; DENV, dengue virus; CHIKV, chikungunya virus.

‡ In-house IgM capture ELISA (following the protocol of the U.S. Centers for Disease Control and Prevention-CDC). Ratios were calculated by dividing the optical density 450nm (O.D.) of the patient’s sample by the O.D. of negative controls. Reference values: <2 = negative; 2–3 = equivocal; >3 = positive.

* CHIKV IgM capture ELISA (Euroimmun). Reference values: <0.8 = negative; ≥0.8 to <1.1 = equivocal; ≥1.1 = positive.

† IgM/IgG capture ELISA (Panbio). Reference values were specific for each kit lot; as different lots were used for the experiments, positive results are labelled as (^POS^) and equivocal results are labelled as (^EQU^); values not labelled as (^POS^) or (^EQU^) are negative.

§ Panbio indirect DENV IgG ELISA. Reference values: <0.9 = negative; 0.9 to 1.1 = equivocal; >1.1 = positive.

+ Indirect DENV IgG ELISA (Panbio) was only performed with acute samples that were non-reactive by the Panbio IgG Capture DENV ELISA.

Among the 22 suspected cases, 16 had PRNT results indicative of an acute infection with ZIKV; the other 6 were discarded (Table 4).

**Table 4:** Molecular and serological results of patients with a suspected Zika virus (ZIKV) infection.

Patient ID	Days post symptoms onset	Lab diagnostic assays													
		qRT-PCR			Serology						PRNT_50_				
					IgM-capture ELISA			IgM/IgG capture ELISA†		IgG-indirect ELISA§+					
		ZIKV	DENV	CHIKV	ZIKV IgM‡	DENV IgM‡	CHIKV IgM*	DENV IgM	DENV IgG	DENV IgG	DENV1	DENV2	DENV3	DENV4	ZIKV
62–0005	4	Neg	Neg	Neg	-	-	-	1.8	0.3	0.02	<20	<20	<20	<20	980.4
	14	-	-	-	18.6	1.4	0.3	2.8	0.3	-	<20	<20	<20	<20	7173
62–0006	1	Neg	Neg	Neg	-	-	-	5.1	17.2	3.3	<20	<20	46.5	244.2	284.1
	15	-	-	-	17.4	6.5	0.2	38.5 ^POS^	57.9 ^POS^	-	<20	<20	272.1	1369	274488
62–0010	2	Neg	Neg	Neg	-	-	-	31.8 ^POS^	13.5	3.2	71.9	<20	28.9	382.1	308.2
	12	-	-	-	18.9	3.3	0.2	95.1 ^POS^	64.1 ^POS^	-	64.2	<20	217.4	2944	5062
**62–0013**	**3**	**Neg**	**Neg**	**Neg**			**-**	**4.7**	**36.5** ^**POS**^	**-**	**<20**	**<20**	**35379**	**1237**	**1021**
	**14**	**-**	**-**	**-**	**4.1**	**2.2**	**0.2**	**5.3**	**45.6** ^**POS**^	**-**	**<20**	**<20**	**100436**	**1906**	**2403**
**62–0024**	**1**	**Neg**	**Neg**	**Neg**	**-**	**-**	**-**	**7.6**	**9.3**	**3.2**	**150.8**	**71.9**	**54.5**	**149.3**	**<100**
	**11**	**-**	**-**	**-**	**7.3**	**1.3**	**0.4**	**7.9**	**9.4**	**-**	**212.9**	**140.9**	**388.4**	**272.2**	**<100**
62–0027	2	Neg	Neg	Neg	-	-	-	42.5 ^POS^	0.3	0.04	<20	<20	<20	<20	<100
	12	-	-	-	11.3	1.4	0.6	29.4 ^POS^	0.3	-	<20	<20	<20	<20	1318
62–0028	1	Neg	Neg	Neg	-	-	-	1.8	5.0	3.3	<20	<20	83.9	248.2	<100
	11	-	-	-	14.3	1.6	0.2	12.5 ^POS^	45.7 ^POS^	-	<20	<20	1282	1387	14092
62–0031	1	Neg	Neg	Neg	-	-	-	7.0	16.9	3.4	<20	<20	213.3	498.1	<100
	11	-	-	-	9.9	1.1	0.3	12.4 ^POS^	62.4 ^POS^	-	<20	<20	1182	20193	1699
62–0039	0	Neg	Neg	Neg	-	-	-	11.9 ^POS^	4.6	3.1	<20	<20	71.7	<20	130.8
	10	-	-	-	5.9	3.0	0.5	15.2 ^POS^	48.4 ^POS^	-	<20	<20	21330	493.4	4027
62–0041	3	Neg	Neg	Neg	-	-	-	5.2	12.6	3.3	<20	<20	113.6	154.8	<100
	19	-	-	-	14.7	1.4	0.3	6.9	38.8 ^POS^	-	<20	<20	653.8	1520	8120
62–0044	2	Neg	Neg	Neg	-	-	-	16.3 ^POS^	0.3	0.03	486.7	763.3	<20	<20	<100
	16	-	-	-	8.8	2.3	0.4	13.4 ^POS^	0.5	-	916.2	863.9	<20	<20	2181
62–0071	1	Neg	Neg	Neg	-	-	-	2.8	12.9	3.4	<20	97.8	138.2	189.2	<100
	18	-	-	-	8.5	1.2	0.3	4.0	48.9 ^POS^	-	<20	92	1004	3251	4174
62–0088	1	Neg	Neg	Pos	-	-	-	5.1	34.1 ^POS^	-	<20	<20	1188	1884	<100
	24	-	-	-	16.6	2.9	5.1	6.8	32.8 ^POS^	-	<20	<20	1167	2070	147.4
**62–0090**	**4**	**Neg**	**Neg**	**Neg**	**-**	**-**	**-**	**4.1**	**36.1** ^**POS**^	**-**	**<20**	**<20**	**318.5**	**321.1**	**133.3**
	**26**	**-**	**-**	**-**	**12.7**	**2.3**	**0.2**	**3.8**	**29.4** ^**POS**^	**-**	**<20**	**<20**	**787.1**	**648.9**	**155.6**
**62–0093**	**3**	**Neg**	**Neg**	**Neg**	**-**	**-**	**-**	**32.9** ^**POS**^	**30.5** ^**POS**^	**-**	**114.4**	**35.4**	**776.6**	**2631**	**598.3**
	**14**	**-**	**-**	**-**	**8.9**	**1.1**	**0.1**	**30.1** ^**POS**^	**25.3** ^**POS**^	**-**	**352.5**	**60.2**	**284.4**	**1268**	**500.9**
62–0100	2	Neg	Neg	Neg	-	-	-	2.1	8.9	2.9	<20	<20	20.3	23.2	111.4
	12	-	-	-	15.2	1.5	0.2	22.6 ^POS^	53.2 ^POS^	-	<20	<20	1327	858.1	23915
**62–0116**	**4**	**Neg**	**Neg**	**Pos**	**-**	**-**	**-**	**9.4**^**EQU**^	**22.6** ^**POS**^	**-**	**183**	**136.8**	**291.2**	**273.2**	**274.9**
	**15**	**-**	**-**	**-**	**3.5**	**1.5**	**4.1**	**7.2**	**23.2** ^**POS**^	**-**	**268.7**	**328.2**	**384.2**	**477.2**	**775.1**
62–0139	1	Neg	Neg	Pos	-	-	-	0.4	40.6 ^POS^	-	<20	<20	699.4	574.2	<100
	11	-	-	-	4.6	1.1	0.4	8.1	38.3 ^POS^	-	<20	<20	579.5	576.5	500
62–0141	2	Neg	Neg	Pos	-	-	-	16.6 ^POS^	27.3 ^POS^	-	<20	<20	895.8	355	171.5
	27	-	-	-	7.0	1.3	0.2	16.7 ^POS^	28.5 ^POS^	-	<20	<20	2547	628.2	600.6
62–0231	0	Neg	Neg	Pos	-	-	-	7.5	53.9 ^POS^	-	41.5	85.9	1987	1953	112.4
	116	-	-	-	5.0	1.6	1.0	7.2	53.7 ^POS^	-	98.1	142.7	1867	3838	804.7
62–0258	1	Neg	Neg	Pos	-	-	-	2.0	17.5	3.7	118.5	87.6	78.1	25.5	556.1
	16	-	-	-	6.6	1.6	5.1	2.3	18.4^EQU^	-	565.8	346.6	68.7	279.9	2056
**62–0263**	**2**	**Neg**	**Neg**	**Neg**	**-**	**-**	**-**	**4.4**	**1.5**	**2.2**	**<20**	**<20**	**<20**	**<20**	**584.7**
	**33**	**-**	**-**	**-**	**3.6**	**1.0**	**3.3**	**1.3**	**1.3**	**-**	**<20**	**<20**	**<20**	**<20**	**1362**

Abbreviations: qRT-PCR, quantitative real time polymerase-chain reaction; ELISA, enzyme-linked immunosorbent assay; IgM, immunoglobulin M; IgG, immunoglobulin G; ZIKV, Zika virus; DENV, dengue virus; CHIKV, chikungunya virus.

‡ In-house IgM capture ELISA (following the protocol of the U.S. Centers for Disease Control and Prevention-CDC). Ratios were calculated by dividing the optical density 450nm (O.D.) of the patient’s sample by the O.D. of negative controls. Reference values: <2 = negative; 2–3 = equivocal; >3 = positive.

* CHIKV IgM capture ELISA (Euroimmun). Reference values: <0.8 = negative; ≥0.8 to <1.1 = equivocal; ≥1.1 = positive.

† IgM/IgG capture ELISA (Panbio). Reference values were specific for each kit lot; as different lots were used for the experiments, positive results are labelled as (^POS^) and equivocal results are labelled as (^EQU^); values not labelled as (^POS^) or (^EQU^) are negative.

§ Panbio indirect DENV IgG ELISA. Reference values: <0.9 = negative; 0.9 to 1.1 = equivocal; >1.1 = positive.

+ Indirect DENV IgG ELISA (Panbio) was only performed with acute samples that were non-reactive by the Panbio IgG Capture DENV ELISA.

In bold: patients that were no longer considered as having a suspected infection with ZIKV after PRNT_50_ interpretation.

### Chikungunya infection

Among the recruited patients, 132 (50.2%) were positive for CHIKV through qRTPCR. The cycle threshold (Ct) values for the qRTPCR were significantly higher for CHIKV (mean Ct = 18.9) compared to ZIKV (mean Ct = 33.5) (*p* <0.0001; *T*-test). Out of the 191 convalescent samples, 95 were positive for CHIKV IgM (49.7%), including 5 participants that were negative for CHIKV through qRTPCR. Based on these results, the number of confirmed CHIKV cases was 132, and 5 cases were considered to be suspected, totaling 137 patients.

Apart from 5 patients (IDs: 62–0088, 62–0139, 62–0141, 62–0231 and 62–0258) that were also considered as having a suspected infection with ZIKV (and are also shown in Table 4), and 2 participants that were later considered as having a suspected infection with DENV, the other samples tested through PRNT showed no positive results for neutralizing antibodies against DENV1-4 or ZIKV (Table 5).

**Table 5:** Molecular and serological results of patients with a confirmed chikungunya virus (CHIKV) infection.

Patient ID	Days post symptoms onset	Lab diagnostic assays													
		qRT-PCR			Serology						PRNT_50_				
					IgM-capture ELISA			IgM/IgG capture ELISA†		IgG-indirect ELISA§+					
		ZIKV	DENV	CHIKV	ZIKV IgM‡	DENV IgM‡	CHIKV IgM*	DENV IgM	DENV IgG	DENV IgG	DENV1	DENV2	DENV3	DENV4	ZIKV
62–0088	1	Neg	Neg	Pos	-	-	-	5.1	34.1 ^POS^	-	<20	<20	1188	1884	<100
	24	-	-	-	16.6	2.9	5.1	6.8	32.8 ^POS^	-	<20	<20	1167	2070	147.4
62–0115	1	Neg	Neg	Pos	-	-	-	3.5	16.8	3.0	<20	<20	24.6	307.6	1230
	16	-	-	-	2.1	7.6	5.6	77.4 ^POS^	18.9^EQU^	-	<20	<20	87.7	1310	1103
62–0116	4	Neg	Neg	Pos	-	-	-	9.4^EQU^	22.6 ^POS^	-	183	136.8	291.2	273.2	274.9
	15	-	-	-	3.5	1.5	4.1	7.2	23.2 ^POS^	-	268.7	328.2	384.2	477.2	775.1
62–0120	1	Neg	Neg	Pos	-	-	-	1.4	3.6	3.1	<20	<20	<20	1020	<100
	11	-	-	-	1.2	3.0	0.3	1.4	3.5	-	<20	<20	<20	1046	<100
62–0126	1	Neg	Neg	Pos	-	-		1.9	7.2	2.9	<20	<20	<20	94.9	<100
	24	-	-	-	1.1	0.8	0.2	1.9	7.2	-	<20	<20	32.2	137	<100
62–0130	2	Neg	Neg	Pos	-	-	-	4.1	29.9 ^POS^	-	<20	<20	1349	1434	623.9
	21	-	-	-	1.5	1.7	4.5	17.9 ^POS^	28.5 ^POS^	-	<20	<20	922.3	673.2	458.7
62–0132	1	Neg	Neg	Pos	-	-	-	2.5	7.6	3.2	<20	<20	401	147.9	<100
	15	-	-	-	1.0	1.1	5.0	1.8	7.1	-	<20	<20	672.6	286.8	<100
62–0136	1	Neg	Neg	Pos	-	-	-	1.6	19.7^EQU^	3.4	<20	<20	1379	1546	<100
	30	-	-	-	2.2	1.1	5.2	1.7	19.7 ^EQU^	-	<20	<20	1626	1645	<100
62–0139	1	Neg	Neg	Pos	-	-	-	6.8	40.6 ^POS^	-	<20	<20	699.4	574.2	<100
	11	-	-	-	4.6	1.1	0.4	8.1	38.3 ^POS^	-	<20	<20	579.5	576.5	500
62–0141	2	Neg	Neg	Pos	-	-	-	16.6 ^POS^	27.3 ^POS^	-	<20	<20	895.8	355	171.5
	27	-	-	-	7.0	1.3	0.2	14.2 ^POS^	28.0 ^POS^	-	<20	<20	2547	628.2	600.6
62–0152	2	Neg	Neg	Pos	-	-	-	1.4	20.5 ^EQU^	3.2	<20	<20	116.4	59.9	<100
	28	-	-	-	0.8	3.2	3.7	0.9	19.9 ^EQU^	-	<20	<20	300	246.2	98.1
62–0158	1	Neg	Neg	Pos	-	-	-	3.9	17.3	3.2	<20	<20	<20	<20	<100
	14	-	-	-	2.8	4.4	5.4	2.9	18.1 ^EQU^	-	<20	<20	<20	<20	<100
62–0172	0	Neg	Neg	Pos	-	-	-	1.7	15.3	3.3	<20	<20	269.8	340.9	721.8
	10	-	-	-	0.9	3.3	4.4	1.4	15.5	-	<20	<20	169.3	316.1	704.3
62–0174	1	Neg	Neg	Pos	-	-	-	1.5	20.6 ^EQU^	3.0	<20	<20	153.2	202.7	293.9
	21	-	-	-	1.0	1.6	4.8	29.3 ^POS^	27.8 ^POS^	-	<20	<20	254.9	266.6	345.4
61–0202	1	Neg	Neg	Pos	-	-	-	3.0	2.9	2.5	<20	<20	251.7	123.6	<100
	18	-	-	-	0.9	1.2	5.1	13.1 ^POS^	3.9	-	<20	26.4	195.2	117.3	<100
62–0231	0	Neg	Neg	Pos	-	-	-	7.5	53.9 ^POS^	-	41.5	85.9	1987	1953	112.4
	116	-	-	-	5.0	1.6	1.0	7.2	53.7 ^POS^	-	98.1	142.7	1867	3838	804.7
62–0258	1	Neg	Neg	Pos	-	-	-	2.0	17.5	3.7	118.5	87.6	78.1	25.5	556.1
	16	-	-	-	6.6	1.6	5.1	2.3	18.4 ^EQU^	-	565.8	346.6	68.7	279.9	2056

Abbreviations: qRT-PCR, quantitative real time polymerase-chain reaction; ELISA, enzyme-linked immunosorbent assay; IgM, immunoglobulin M; IgG, immunoglobulin G; ZIKV, Zika virus; DENV, dengue virus; CHIKV, chikungunya virus.

‡ In-house IgM capture ELISA (following the protocol of the U.S. Centers for Disease Control and Prevention-CDC). Ratios were calculated by dividing the optical density 450nm (O.D.) of the patient’s sample by the O.D. of negative controls. Reference values: <2 = negative; 2–3 = equivocal; >3 = positive.

* CHIKV IgM capture ELISA (Euroimmun). Reference values: <0.8 = negative; ≥0.8 to <1.1 = equivocal; ≥1.1 = positive.

† IgM/IgG capture ELISA (Panbio). Reference values were specific for each kit lot; as different lots were used for the experiments, positive results are labelled as (^POS^) and equivocal results are labelled as (^EQU^); values not labelled as (^POS^) or (^EQU^) are negative.

§ Panbio indirect DENV IgG ELISA. Reference values: <0.9 = negative; 0.9 to 1.1 = equivocal; >1.1 = positive.

+ Indirect DENV IgG ELISA (Panbio) was only performed with acute samples that were non-reactive by the Panbio IgG Capture DENV ELISA.

### Dengue infection

No patients were positive for DENV though qRTPCR, and only one was positive for DENV NS1 Ag; this was the only patient with a confirmed DENV diagnosis.

Using the Panbio capture assays, the percentages of samples positive for anti-DENV IgM and IgG in the acute phase (n = 263) were 11.8% and 33.1%, respectively. In the convalescent phase, the percentages of samples positive for anti-DENV IgM and IgG were 17.3% and 41.4%, respectively, among the patients who had a convalescent sample (n = 191). Out of the 191 patients who had a convalescent sample, 16 seroconverted to anti-DENV IgM. Of these, 12 (75.0%) were positive for ZIKV through qRTPCR or anti-ZIKV IgM assays, and the remaining 4 (25.0%) were positive for CHIKV through qRTPCR and anti-CHIKV IgM. The number of patients who seroconverted to anti-DENV IgG was 19 and, among these, 16 (84.2%) were positive for ZIKV through qRTPCR or anti-ZIKV IgM assays, and 1 (5.3%) (different from the patients who were positive for ZIKV) was positive for CHIKV through qRTPCR and anti-CHIKV IgM.

The percentages of acute samples that were negative for anti-DENV IgG through the Panbio Capture ELISA and Indirect ELISA were 54.4% and 4.9%, respectively. The percentage of acute samples with an equivocal result was 12.5% for the capture ELISA and 0.8% for the indirect test.

When convalescent samples were assayed using the in-house IgM MAC-ELISA (CDC protocol), which uses DENV and ZIKV antigens in parallel, 4 out of 191 (2.1%) were positive for DENV and were considered as DENV suspected. Out of these 4, 2 remained as DENV suspected and 2 were discarded based on PRNT results. Apart from these, 3 other participants were considered as having a suspected DENV infection, based on PRNT results (IDs: 62–0030, 62–0033, and 62–0096) (Table 6).

**Table 6:** Molecular and serological results of patients who did not have a confirmed or suspected infection with Zika (ZIKV) or chikungunya (CHIKV) viruses.

Patient ID	Days post symptoms onset	Lab diagnostic assays													
		qRT-PCR			Serology						PRNT_50_				
					IgM-capture ELISA			IgM/IgG capture ELISA†		IgG-indirect ELISA§+					
		ZIKV	DENV	CHIKV	ZIKV IgM‡	DENV IgM‡	CHIKV IgM*	DENV IgM	DENV IgG	DENV IgG	DENV1	DENV2	DENV3	DENV4	ZIKV
62–0002	2	Neg	Neg	Neg	-	-	-	2.9	0.4	0.07	<20	<20	<20	<20	<100
	12	-	-	-	1.1	1.2	0.3	3.5	0.3	-	<20	<20	<20	<20	<100
62–0025	2	Neg	Neg	Neg	-	-	-	24.8 ^POS^	32.2 ^POS^	-	<20	<20	281.1	785.3	745.2
	12	-	-	-	1.5	1.3	0.1	16.9 ^POS^	28.3 ^POS^	-	<20	<20	792.3	1016	1509
62–0029	2	Neg	Neg	Neg	-	-	-	3.9	7.4	3.3	<20	<20	3139	280.9	1917
	13	-	-	-	19.2	16.7	0.2	4.2	28.2 ^POS^	-	<20	<20	1282	1046	1937
62–0030	2	Neg	Neg	Neg	-	-	-	5.8	11.2	3.2	<20	<20	2297	176.5	666.2
	16	-	-	-	2.0	1.3	0.4	7.6	36.8 ^POS^	-	<20	<20	12721	1453	4255
62–0033	2	Neg	Neg	Neg	-	-	-	3.2	28.5 ^POS^	-	<20	<20	2777	269	1922
	12	-	-	-	9.7	8.7	0.2	3.1	30.4 ^POS^	-	<20	38.6	53735	296.8	1196
62–0043	2	Neg	Neg	Neg	-	-	-	4.3	6.0	3.0	<20	<20	185.4	333	115
	15	-	-	-	1.9	2.2	0.3	4.3	5.9	-	<20	<20	182.1	324.5	211.6
62–0046	1	Neg	Neg	Neg	-	-	-	3.4	19.2 ^EQU^	3.3	<20	<20	895.1	414.9	<100
	13	-	-	-	1.4	1.0	0.3	3.5	20.4 ^EQU^	-	<20	<20	841.1	1511	<100
62–0051	3	Neg	Neg	Neg	-	-	-	2.9	0.7	1.9	<20	<20	<20	<20	1200
	14	-	-	-	1.1	0.9	0.2	3.0	0.6	-	<20	<20	<20	<20	2288
62–0055	0	Neg	Neg	Neg	-	-	-	5.5	16.0	3.4	<20	<20	223.1	317.8	<100
	31	-	-	-	1.1	0.8	0.3	4.5	16.4	-	<20	<20	190.1	680	<100
62–0057	2	Neg	Neg	Neg	-	-	-	9.4 ^EQU^	27.0 ^POS^	-	<20	<20	365.7	543.1	1024
	20	-	-	-	6.1	4.0	0.1	7.3	25.9 ^POS^	-	<20	<20	351.9	403.7	809.3
62–0058	2	Neg	Neg	Neg	-	-	-	8.3	42.2 ^POS^	-	<20	<20	276.7	651.4	<100
	16	-	-	-	1.0	0.8	1.0	8.1	42.5 ^POS^	-	<20	<20	611.2	791	<100
62–0062	0	Neg	Neg	Neg	-	-	-	5.7	12.5	3.3	<20	<20	211.2	178.6	<100
	31	-	-	-	0.9	0.8	0.3	5.6	12.7	-	<20	<20	292.8	513.9	<100
62–0067	1	Neg	Neg	Neg	-	-	-	5.5	14.8	3.4	<20	<20	275.1	701.4	157.6
	28	-	-	-	1.1	0.7	0.4	5.3	10.2	-	<20	<20	129.3	658.3	206.5
62–0070	1	Neg	Neg	Neg	-	-	-	5.6	44.6 ^POS^	-	<20	<20	1252	1346	145.7
	24	-	-	-	1.0	1.1	0.3	4.8	42.4 ^POS^	-	<20	<20	1025	4008	1262
62–0075	1	Neg	Neg	Neg	-	-	-	9.9 ^EQU^	44.9 ^POS^	-	24.4	<20	627.3	1672	<100
	13	-	-	-	1.8	0.9	0.3	9.6 ^EQU^	44.3 ^POS^	-	66.3	52	746	3171	255.5
62–0079	3	Neg	Neg	Neg	-	-	-	2.0	0.2	0.03	<20	<20	<20	<20	<100
	13	-	-	-	1.1	0.9	0.5	1.0	0.2	-	<20	<20	<20	<20	<100
62–0084	0	Neg	Neg	Neg	-	-	-	1.8	5.2	3.2	<20	<20	346.4	145.4	<100
	10	-	-	-	1.0	0.9	0.2	1.7	5.5	-	<20	<20	371	143	112.3
62–0089	1	Neg	Neg	Neg	-	-	-	3.2	7.4	3.2	<20	<20	93.2	120.9	<100
	12	-	-	-	1.4	1.1	0.1	3.0	8.3	-	<20	<20	173	95.1	<100
62–0092	1	Neg	Neg	Neg	-	-	-	24.8 ^POS^	0.2	0.02	<20	<20	<20	<20	<100
	12	-	-	-	1.0	1.0	0.3	23.3 ^POS^	0.2	-	<20	<20	<20	<20	<100
62–0094	2	Neg	Neg	Neg	-	-	-	3.4	3.5	3.3	<20	<20	227.3	304.4	<100
	15	-	-	-	1.1	0.9	0.3	3.4	3.6	-	<20	<20	353.4	502.5	<100
62–0096	2	Neg	Neg	Neg	-	-	-	2.8	5.2	3.0	<20	<20	166.2	61.8	<100
	12	-	-	-	1.0	1.3	0.2	2.9	5.5	-	<20	<20	116.6	613.8	<100
62–0105	0	Neg	Neg	Neg	-	-	-	5.3	23.8 ^POS^	-	<20	<20	721.5	819.9	<100
	10	-	-	-	2.2	2.2	0.2	5.4	24.4 ^POS^	-	<20	<20	1158	930	<100
62–0121	2	Neg	Neg	Neg	-	-	-	5.8	14.2	3.4	<20	<20	93.4	91.7	<100
	13	-	-	-	1.1	0.7	0.4	5.7	13.5	-	<20	<20	85.7	114	<100
62–0124	2	Neg	Neg	Neg	-	-	-	1.3	18.3 ^EQU^	3.4	21.7	<20	94.2	88.8	<100
	18	-	-	-	1.2	1.1	0.4	1.3	17.2	-	55	<20	162.2	155.1	<100
62–0142	2	Neg	Neg	Neg	-	-	-	8.3	0.3	1.2	<20	<20	<20	<20	461.5
	34	-	-	-	2.7	1.1	0.5	8.6	0.4	-	<20	<20	<20	<20	1052
62–0179	1	Neg	Neg	Neg	-	-	-	15.7 ^POS^	9.6	3.0	<20	<20	<20	61.6	<100
	51	-	-	-	0.9	1.2	0.3	12.9 ^POS^	8.7	-	<20	<20	54.3	82.2	<100
62–0212	3	Neg	Neg	Neg	-	-	-	6.3	17.5	3.2	<20	<20	560.7	429.7	<100
	40	-	-	-	1.0	1.0	0.2	5.5	16.8	-	<20	<20	860.9	533.2	<100
62–0233	3	Neg	Neg	Neg	-	-	-	2.0	13.5	3.1	<20	<20	122.8	75.8	<100
	49	-	-	-	1.1	0.8	0.4	2.1	14.1	-	<20	<20	159.8	70.4	<100
62–0248	1	Neg	Neg	Neg	-	-	-	3.3	21.6 ^EQU^	3.2	<20	<20	170.8	310.1	532.7
	18	-	-	-	2.8	1.0	0.3	2.7	19.3 ^EQU^	-	<20	<20	428.7	334.1	1207

Abbreviations: qRT-PCR, quantitative real time polymerase-chain reaction; ELISA, enzyme-linked immunosorbent assay; IgM, immunoglobulin M; IgG, immunoglobulin G; ZIKV, Zika virus; DENV, dengue virus; CHIKV, chikungunya virus.

‡ In-house IgM capture ELISA (following the protocol of the U.S. Centers for Disease Control and Prevention-CDC). Ratios were calculated by dividing the optical density 450nm (O.D.) of the patient’s sample by the O.D. of negative controls. Reference values: <2 = negative; 2–3 = equivocal; >3 = positive.

* CHIKV IgM capture ELISA (Euroimmun). Reference values: <0.8 = negative; ≥0.8 to <1.1 = equivocal; ≥1.1 = positive.

† IgM/IgG capture ELISA (Panbio). Reference values were specific for each kit lot; as different lots were used for the experiments, positive results are labelled as (^POS^) and equivocal results are labelled as (^EQU^); values not labelled as (^POS^) or (^EQU^) are negative.

§ Panbio indirect DENV IgG ELISA. Reference values: <0.9 = negative; 0.9 to 1.1 = equivocal; >1.1 = positive.

+ Indirect DENV IgG ELISA (Panbio) was only performed with acute samples that were non-reactive by the Panbio IgG Capture DENV ELISA.

**Fig 3:**
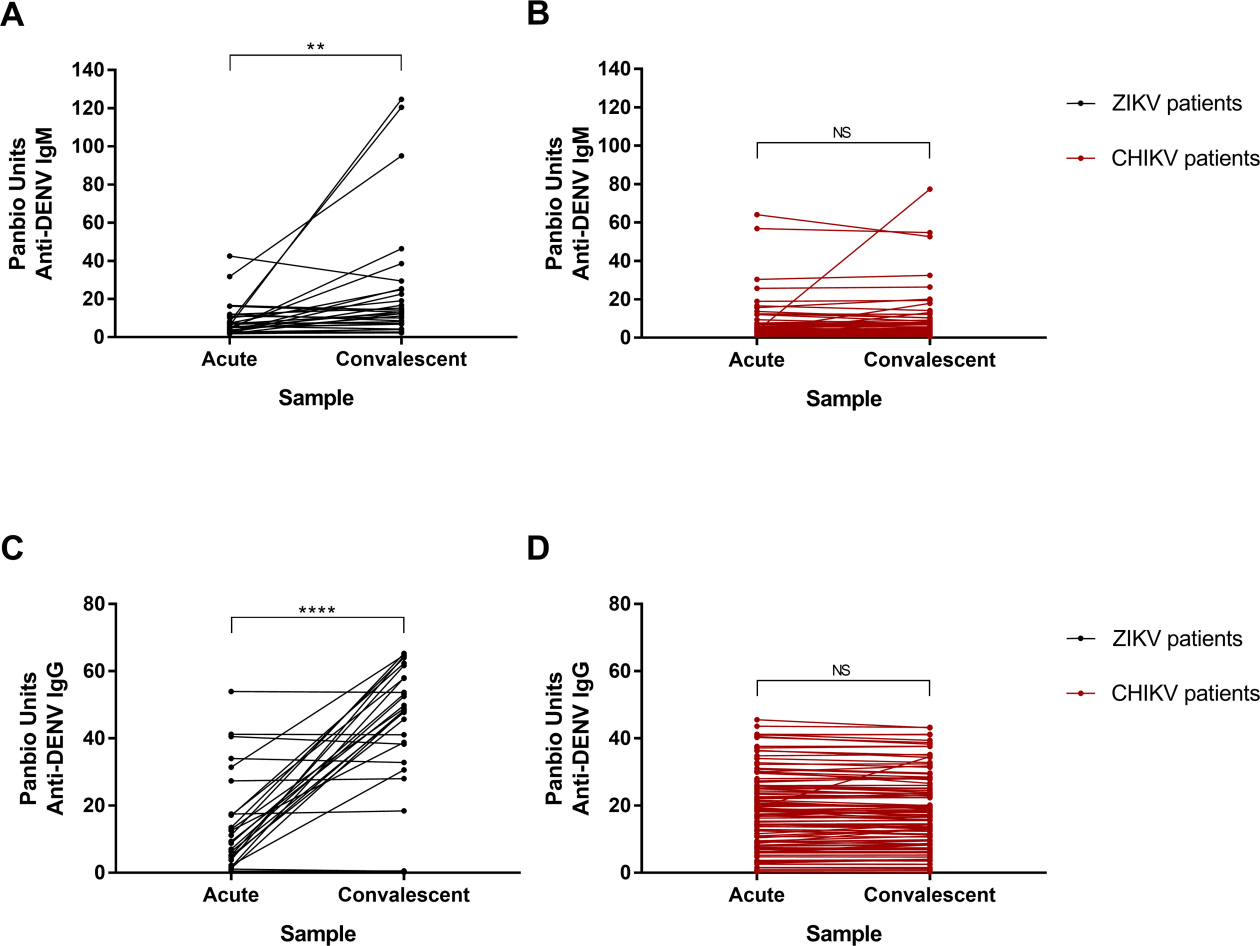
Dengue virus (DENV) serological assays. (A) Amount of IgM reactive to DENV in the acute and convalescent samples of participants infected with Zika virus (ZIKV). (B) Amount of IgM reactive to DENV in the acute and convalescent samples of participants infected with chikungunya virus (CHIKV). (C) Amount of IgG reactive to DENV in the acute and convalescent samples of participants infected with Zika virus (ZIKV). (D) Amount of IgG reactive to DENV in the acute and convalescent samples of participants infected with chikungunya virus (CHIKV). ELISA optical density was converted in Panbio units. Asterisks reflect the level of significance between groups after paired *T*-test was performed: **p <0.05, ****p <0.0001; NS = non-significant.

Among the patients with a confirmed ZIKV infection, 4.0% and 61.5% were positive for DENV IgM in the acute and convalescent phases, respectively, and 20.0% and 69.2% were positive for DENV IgG in the acute and convalescent phases, respectively, using the Panbio capture assays. The levels of anti-DENV IgM and IgG in ZIKV-positive samples were significantly higher in the convalescent phase than in the acute phase (*p* <0.05 for IgM and *p* <0.0001 for IgG; *T*-test) (Fig 3).

Among the patients who tested positive for CHIKV through qRTPCR, 9.0% and 8.8% were positive for anti-DENV IgM in the acute and convalescent phases, respectively, and 36.4% and 37.6% were positive for anti-DENV IgG in the acute and convalescent phases, respectively, using the Panbio capture assays. Anti-DENV IgM and IgG did not differ between the acute and convalescent phases in CHIKV-positive patients (*p* >0.05; *T*-test) (Fig 3).

### Non-ZIKV, non-CHIKV

Of the 29 patients that were not considered as having an infection with ZIKV or CHIKV and were tested by PRNT, three patients were diagnosed as having a suspected infection with DENV (cited above) and the remaining were indeterminate (Table 6).

### Spatiotemporal distribution of ZIKV and CHIKV cases

**Fig 4:**
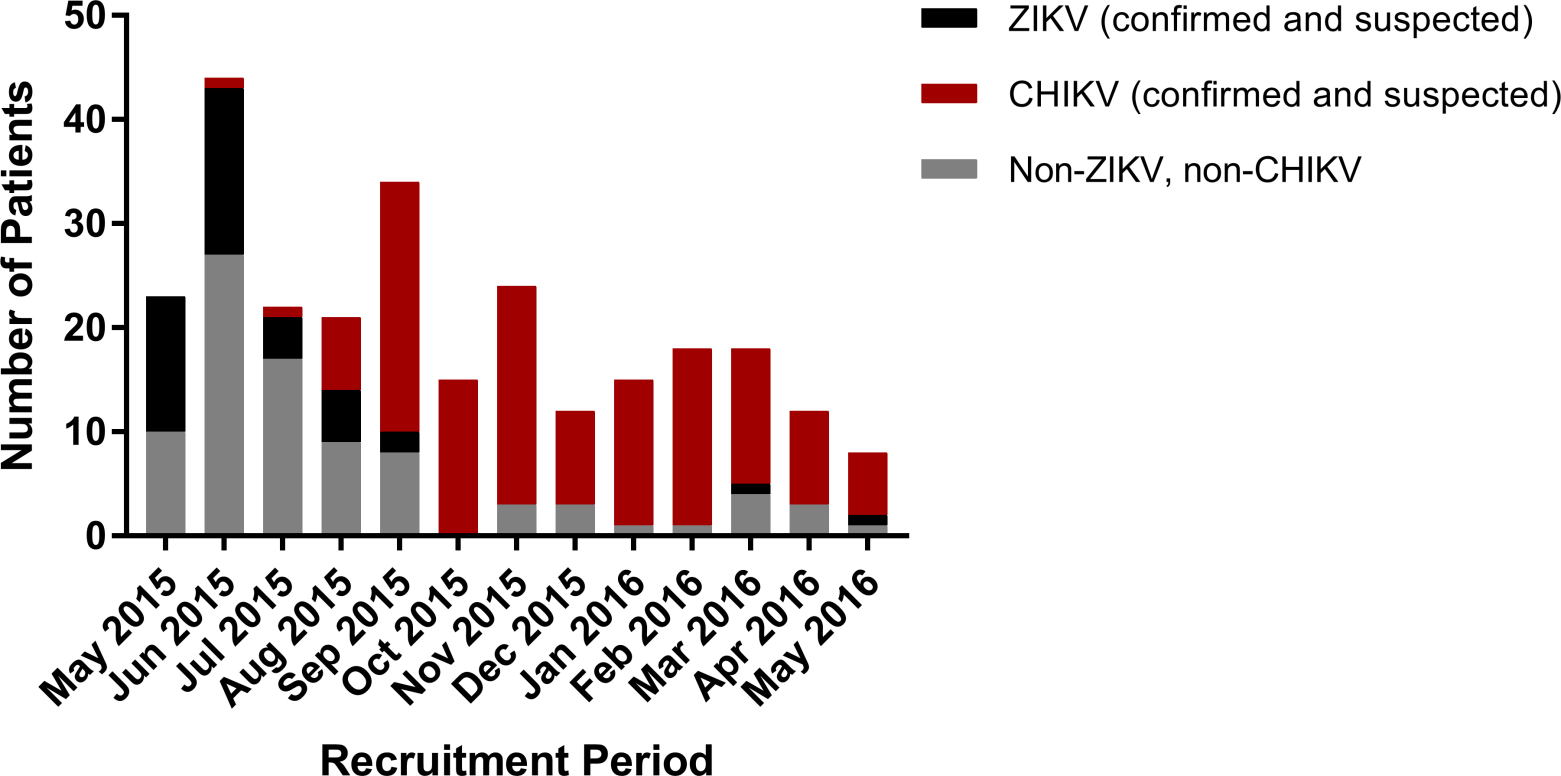
Zika and chikungunya infections. Absolute number of participants infected with Zika virus (ZIKV) and chikungunya virus (CHIKV), and those not infected with either virus (Non-ZIKV, non-CHIKV), per month.

The temporal distribution of cases showed that confirmed and suspected ZIKV-infected patients were concentrated in the first four months of recruitment (May-August 2015), reaching 52.0% positivity in May 2015 and decreasing gradually to 6.3% in September. After that, one case of ZIKV was detected in March 2016 and one in May 2016 (Fig 4).

The first CHIKV-positive case was detected in June 2015 (2.3%) and cases started to rise in August 2015 (35%). Throughout the remainder of the study period, the detection of CHIKV-positive patients occurred in all months, with an average of 84.7% of infection per month among the recruited patients from August 2015 to May 2016 (Fig 4).

**Fig 5:**
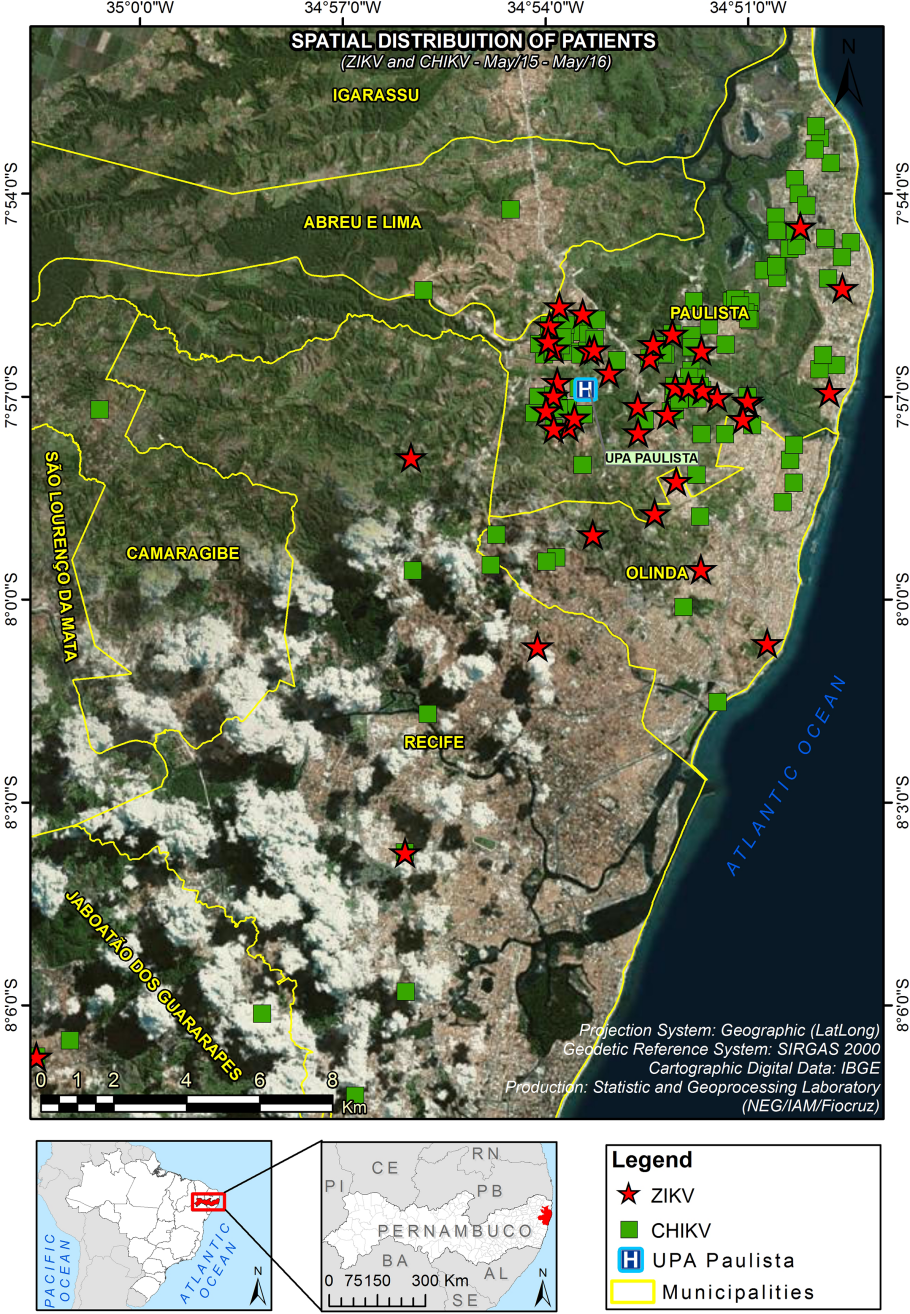
Spatial distribution of participants infected with Zika virus (ZIKV) or chikungunya virus (CHIKV) in the study area, from May 2015 to May 2016. The distribution of patients was superimposed with a satellite photograph of the region. Confirmed and suspected cases of Zika virus (ZIKV) or chikungunya virus (CHIKV) infection were used in the analysis. Red star: Zika case; green square: chikungunya case. The geodetic reference system SIRGAS2000 (Geocentric Reference System for the Americas) was the coordinated system used to represent geometric or physical terrestrial characteristics (http://www.ibge.gov.br/english/geociencias/geodesia/pmrg/faq.shtm#1).

The majority of ZIKV- and CHIKV-positive cases were distributed in urbanized areas surrounding the UPA-Paulista, and only a few cases were spread farther from the UPA and in neighboring cities (Abreu e Lima, Camaragibe, Jaboatão dos Guararapes, Recife and Olinda) (Fig 5).

**Fig 6:**
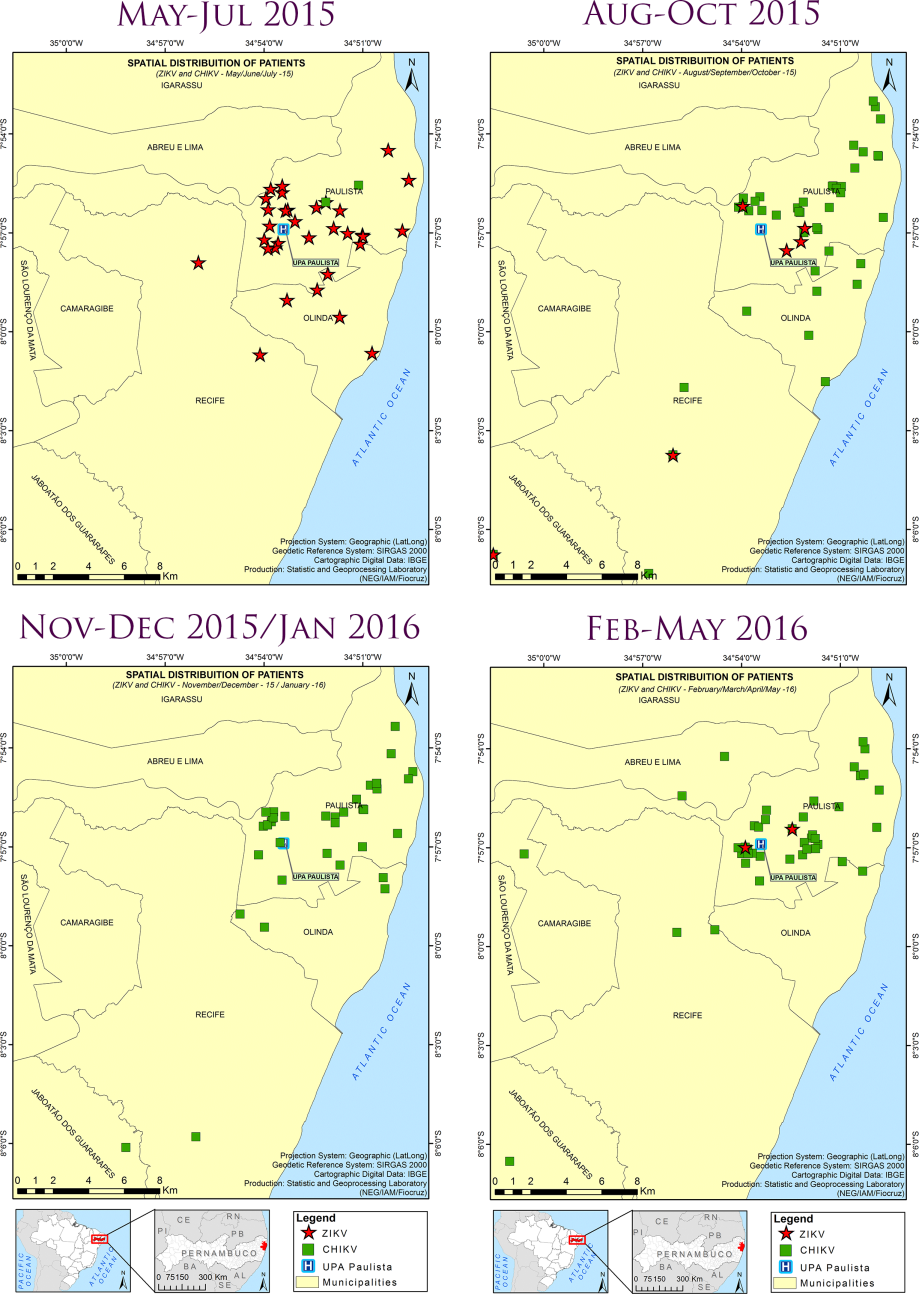
Spatiotemporal distribution of patients infected with Zika virus (ZIKV) or chikungunya virus (CHIKV), from May 2015 to May 2016, in the study area. Spatiotemporal distribution of Zika virus (ZIKV) or chikungunya virus (CHIKV) infection cases in periods of three to four months, from May 2015 to May 2016. Confirmed and suspected cases were used in the analysis. Red star: Zika case; green square: chikungunya case. The geodetic reference system SIRGAS2000 (Geocentric Reference System for the Americas) was the coordinated system used to represent geometric or physical terrestrial characteristics (http://www.ibge.gov.br/english/geociencias/geodesia/pmrg/faq.shtm#1).

**Fig 7:**
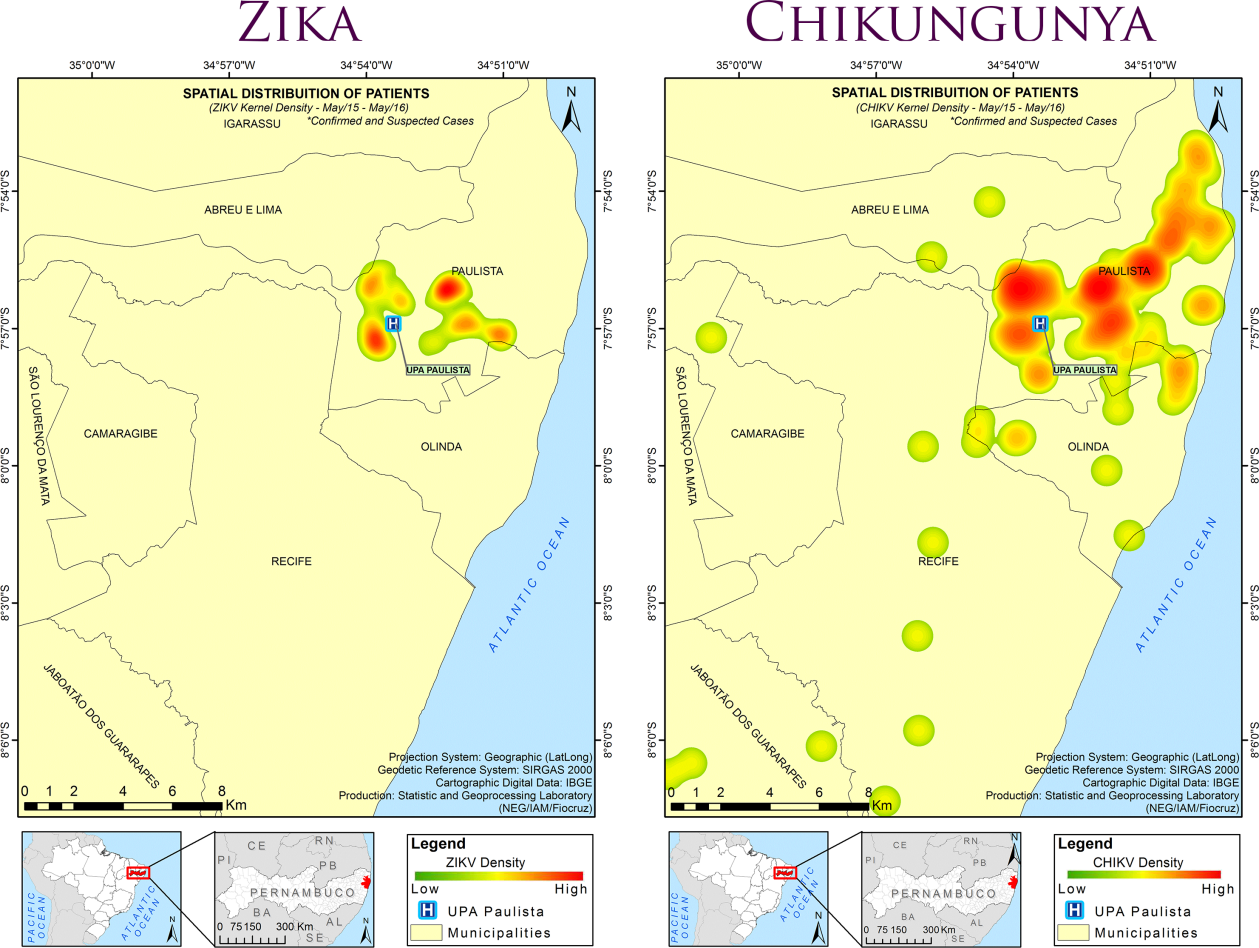
Hotspots of the distribution of participants infected with Zika virus (ZIKV) or chikungunya virus (CHIKV), from May 2015 to May 2016, in the study area. Kernel density map of the distribution of patients with a confirmed or suspected infection with Zika virus (ZIKV) of chikungunya virus (CHIKV). The geodetic reference system SIRGAS2000 (Geocentric Reference System for the Americas) was the coordinated system used to represent geometric or physical terrestrial characteristics (http://www.ibge.gov.br/english/geociencias/geodesia/pmrg/faq.shtm#1).

The spatial distribution of 3- to 4-month periods showed some clustering of chikungunya cases, while this was not observed for Zika cases (Fig 6). Hotspots of Zika and chikungunya cases were identified by the Kernel density analysis (Fig 7).

### Co-infections

Two cases of co-infection with ZIKV and CHIKV were detected through qRTPCR; both patients were recruited in August 2015. The number of co-infections based on serological assays was not assessed.

### Proportions of women and men infected with ZIKV or CHIKV

The percentage of women in the ZIKV-infected group (64%, 95%-CI: 48%-78%) was higher compared to the CHIKV-infected group (39%, 95%-CI: 31%-48%). The test of proportions was statistically significant at the 5% level (*p* = 0.005), also when excluding the two persons (1 woman, 1 man) who were co-infected with ZIKV and CHIKV (*p* = 0.004) (Fig 1).

## Discussion

In this study, most (60.0%) of the patients with acute fever suspected of an arboviral disease had a confirmed infection with ZIKV or CHIKV. Serological assays corroborated qRTPCR data and also identified probable cases of infections. Unexpectedly, molecular and serological assays indicated a very low proportion of DENV infections in an area that has been endemic for this virus for the past several years, with circulation of all four serotypes (DENV1-4) [1–3]. These data reflect the dimensions of the Zika and chikungunya epidemics in the study area in 2015–2016. Still, in 2015 a very high number of dengue cases were officially notified in Pernambuco; while 9,729 cases were notified in 2014, 92,395 cases were notified in 2015, mainly in the first half of the year [3]. Accordingly, the number of notified dengue cases at the UPA-Paulista increased from 186 in 2014 to 1,326 in 2015, also in the first half of the year (UPA-Paulista, internal document). It is possible though that the sharp increase in the number of notified dengue cases in 2015 was greatly biased by cases of Zika infections that were occurring in the region at the time, as has been suggested by other authors [28]. Our results corroborate this assumption. A recent case-control study on Zika in Recife reported that 64.0% of the study group had positive serology for ZIKV [29], strengthening the notion that circulation of ZIKV in Pernambuco in 2015 was very high. The estimated number of ZIKV infections in Brazil in 2015 was calculated based on the number of cases that were reported and then discarded as dengue cases, as well as on international projections of Zika infections [13]. The data presented here suggests that notified dengue cases (not confirmed, not discarded) in 2015 in areas where ZIKV was circulating should also be considered in these estimates.

The temporal distribution of cases indicated that we captured the tail end of the Zika epidemic in 2015 in Recife. After a gradual decrease in ZIKV cases from May to September 2015, no more patients infected with the virus were detected for 5 consecutive months (from October 2015 to February 2016) and only two patients were positive for ZIKV in March and May 2016 (one in each month). Concomitant to a decrease in Zika cases, the number of chikungunya cases started to rise in August 2015. Subsequently, the percentage of patients infected with CHIKV was very high, even during the dry season (September-November 2015 [30]). These data suggest the displacement of ZIKV by CHIKV in the study area, possibly caused by virus competition in humans and mosquito vectors, and other factors such as acquired immunity to ZIKV in the human population and the high transmission efficiency of CHIKV. Displacement patterns have been observed for distinct DENV serotypes in endemic areas [31, 32] and may occur with distinct arboviruses sharing the same hosts. We also observed that, in our strategy, chikungunya cases were detected 5 months before official cases started to be notified in Paulista-PE, indicating that the screening strategy adopted here was highly efficient in detecting new arboviral infections in the beginning of an epidemic.

The efficiency of CHIKV transmission was also reflected by the lower Ct values of CHIKV obtained through qRTPCR in comparison to ZIKV, indicating a higher viremia of the former in the patients. A higher viral load of CHIKV compared to ZIKV in patient samples has been previously reported [33] and high Ct values (reflecting a lower amount of virus in the sample tested) from ZIKV-positive samples have been shown in several studies [22, 34].

Zika and chikungunya cases were mainly distributed in urbanized areas around UPA-Paulista, except for a few cases near forested areas. However, while chikungunya cases appeared to form clusters, this was not observed for Zika. Cluster formation was strengthened by the Kernel density-based analysis, which showed strong hotspots of chikungunya cases, and may further be indicative of the transmission efficiency of this virus by mosquitoes. The identification of hotpots of human cases may help to guide control activities against these diseases, including those aimed at eliminating the vectors.

Reported rates of ZIKV/CHIKV co-infections in humans are, in general, low, ranging from 0% to 4.6% [33, 35, 36]. Interestingly, *Ae*. *aegypti* mosquitoes that are co-infected with ZIKV and CHIKV are capable of transmitting both viruses [37, 38], contradicting in a way the idea of competition. However, viral load of ZIKV in mammalian cells, mosquito cells and whole mosquitoes decrease upon co-infection with ZIKV and CHIKV [37, 38]. In the field, where several factors play a role in the transmission cycle of an arbovirus, a lower viral load of a virus due to co-infection may be relevant. Also, in our opinion, co-infection studies in the vector should try to simulate, in the artificial blood meals, the natural lower viremia of ZIKV compared to CHIKV observed in humans, as this may significantly affect mosquito co-infection outcomes. These facts and the immune status of the local population towards an arbovirus before the introduction of a second arbovirus, may substantially minimize human co-infections. Here, only two (0.8%) cases of confirmed co-infection with ZIKV and CHIKV were detected and both occurred in August 2015, when the proportions of infection of both viruses was similar (25–35%). After the number of CHIKV increased, no more co-infections were detected. Overall, our results indicate that the introduction of CHIKV in the area helped to suppress the circulation of ZIKV and that both viruses helped to suppress the circulation of DENV. This is in accordance with the idea that arboviruses that share the same invertebrate and vertebrate hosts within a limited geographic area circulate in cycles where one of them predominates, and that several factors dictate their success, including viral competition and the immune status towards these pathogens in the human population.

A female ratio bias among ZIKV-infected patients has been previously described in Brazil, Puerto Rico, Nicaragua, the U.S. and France, and may strengthen the role of sexual transmission in Zika epidemiology (Reviewed in [39]). However, this is the first study showing that CHIKV-infected patients from the same cohort showed a male bias. It is important to mention that by the time ZIKV-infected patients were enrolled, neither the study team nor the patients knew that ZIKV was circulating in the Recife region, as the first detection of ZIKV infection in Brazil occurred in Bahia State in May 2015 [40]. Additionally, the association of ZIKV-infection to congenital Zika syndrome was not recognized until October 2015 [11]. Thus, the possibility of care-seeking bias from ZIKV-infected women being more prone to go to a health clinic due to concerns on the reproductive health may be ruled out. The male bias in CHIKV-infected patients is also interesting and merits further investigation.

Cross-reactive antibodies against DENV and ZIKV are considered a serious issue in serological assays aimed at identifying infection by either virus [22, 41–43]. Here, although IgM and IgG reactive to DENV were relatively high in the early convalescent sample of recruited patients using the Panbio capture kits, our data strongly indicate that high cross-reactivity occurred with patients who were infected with ZIKV. First, antibodies reactive to DENV in ZIKV-infected patients were significantly higher in the convalescent phase than in the acute phase, indicating that antibodies developed against ZIKV during an active infection cross-reacted with DENV antigens in the assays; importantly, the same was not observed for CHIKV-infected samples. Second, when convalescent samples were tested with the CDC ZIKV IgM MAC-ELISA, which assays ZIKV and DENV antigens in parallel, only 2.1% of the convalescent samples were positive, compared to 17.3% using the Panbio capture assay. Lastly, most of the patients who seroconverted to anti-DENV IgM or IgG, when tested with the Panbio capture kits, were positive for ZIKV and only one of these was positive for DENV NS1.

An important data obtained from serological assays using DENV antigens was that the percentage of reactive anti-DENV IgG in the acute samples was relatively low (33%) when the Panbio Capture ELISA was used. However, when samples that were non-reactive in the IgG capture ELISA were tested by the Panbio Indirect ELISA, the proportion of patients with IgG reactive to DENV in the acute phase increased to 95%. This was more expected as the region has been endemic for DENV for decades. The indirect test seems to be more appropriate for epidemiological analyses due to a lower cut-off which more reliably reflects the status of patients in regards to a past infection with DENV. These results show the importance of choosing the appropriate test to correctly answer a study question, as the number of DENV-naïve patients would have been greatly overestimated if the results of the capture assay were analyzed for this purpose. However, in our case it is still possibe that some acute samples were positive in this test due to a previous (but recent) infection with ZIKV.

The PRNTs were very important to validate the Zika cases. Still, a few samples were indeterminate in regards to their exposure to DENV or ZIKV even after this assay was performed (for instance, participants 62–0070 and 62–0075), highlighting the difficulty in the interpretation of serological assays in samples of people living in areas where these viruses co-circulate.

Finally, in this study we concluded that: 1) cross-reactivity of serological assays must be thoroughly evaluated by local health authorities in areas with simultaneous circulation of multiple arboviruses. Besides the need for the development and implementation of more precise serological methods for DENV and ZIKV, there should also be efforts to implement point-of-care molecular assays for arbovirus detection in endemic countries as these are the most reliable tests for laboratory diagnosis of these diseases; 2) the distribution of Zika and chikungunya cases in the same urban areas, and the displacement of ZIKV by CHIKV, suggests the involvement of the same urban vectors in their transmission; however, some differences in the distribution pattern (e.g. cluster formation of chikungunya cases) may indicate differences in the transmission efficiency between the viruses and the involvement of other transmission modes in the case of ZIKV (e.g., sexual transmission); 3) the strategy implemented in our study to screen for patients with a clinical suspicion of an arboviral infection in the first three days of acute symptoms proved to be efficient in detecting these diseases early in the epidemics. If this strategy is adopted in health centers, control and prevention strategies may be implemented earlier to minimize disease transmission; and 4) simple and more precise laboratory and clinical markers of arboviral diseases are urgently needed as they would greatly improve case management in endemic countries.
